# Prehabilitation for patients undergoing neoadjuvant treatment: results of a single-center pilot study using an integrative medicine day clinic

**DOI:** 10.3389/fonc.2026.1913126

**Published:** 2026-09-14

**Authors:** Svenja Klaus-Karwisch, Stephanie Otto, Paul Georg Werthmann, Ann-Kathrin Lederer, André Leopold Mihaljevic, Fynn Treiber, Lena Cihlar, Keanu Leins, Benjamin Mayer, Klaus Kramer

**Affiliations:** 1 Section Integrative Medicine, Department of General and Visceral Surgery, Ulm University Medical Center, Ulm University, Ulm, Germany; 2 Comprehensive Cancer Center Ulm (CCCU), Ulm University Hospital, Ulm, Germany; 3 Department of Gynaecology and Obstetrics, Ulm University Hospital, Ulm, Germany; 4 Department of Internal Medicine I, Ulm University Hospital, Ulm, Germany; 5 Department of Internal Medicine III, Ulm University Hospital, Ulm, Germany; 6 Fascia Research Society (FRS), Burnsville, MN, United States; 7 Department of General, Visceral and Transplantation Surgery, University Hospital Tuebingen, Tübingen, Germany; 8 Institute of Epidemiology and Medical Biometry, University Ulm, Ulm, Germany; 9 University Ulm, Ulm, Germany

**Keywords:** integrative oncology, mind-body medicine, multimodal prehabilitation, neoadjuvant treatment, supportive care

## Abstract

**Introduction:**

Oncological prehabilitation aims to strengthen cancer patients physically and mentally before treatment, yet it is not standard care. The Prehabilitation through an Integrative Medicine Day Clinic (PRIME-DC) study examined the feasibility of a multimodal program during neoadjuvant therapy prior to surgery.

**Material and methods:**

In this single-arm, mono-center interventional feasibility trial, patients attended a closed-group program once weekly for eight weeks (6.5-hour sessions) scheduled across the neoadjuvant treatment period. The curriculum integrated physical exercise, nutritional and naturopathic counseling, and Mind-Body Medicine practices. The primary outcome was program adherence, defined by attendance rate. Secondary outcomes included patient-reported measures of quality of life, anxiety, depression, fatigue, sleep, mindfulness, and physical fitness tests assessed at baseline and post-intervention. Patient satisfaction were evaluated at program completion.

**Results:**

Twenty-three of the 41 patients who were assessed for eligibility were enrolled, with an adherence rate of 69%. An exploratory within-group improvement in the 1-minute sit-to-stand test was observed from baseline to post-intervention (V1: 26.00 (22.00/34.00), V10a: 27.00 (24.99/34.00), 95% CI = 1.000 – 6.000, p = 0.02, r = 0.66), while other functional and patient-reported outcomes showed no statistically detectable within-group changes (p > 0.05). No study-related adverse events occurred. Patient satisfaction was assessed using a 17-item Likert scale (0–4). Overall, patients reported high levels of satisfaction (range: 0.00 - 1.88).

**Conclusion:**

This pilot feasibility study provides preliminary evidence that participation in an integrative prehabilitation day-clinic program may be associated with the preservation of physical and mental health during neoadjuvant treatment. The findings indicate good feasibility and acceptability, providing a strong rationale for further investigation in larger, controlled trials.

**Clinical trial registration:**

https://drks.de/search/de/trial/DRKS00028126, identifier DRKS00028126.

## 1 Introduction

Neoadjuvant therapy (NAT) plays a crucial role in many solid tumors to optimize surgical outcomes and improve long-term prognosis (1–5). However, across multiple tumor entities, patients typically experience a decline in health-related Quality of Life (QoL) and physical fitness during NAT, reflecting shared treatment-related and perioperative challenges (6, 7). Furthermore, reduced postoperative fitness and QoL are associated with poorer surgical outcomes and delayed recovery in patients undergoing oncologic surgery (8, 9). To address this challenging period, prehabilitation was established. Unlike traditional rehabilitation, which focuses on recovery after treatment-related declines, prehabilitation aims to optimize a patient’s physical, nutritional, and mental health status before surgery or during NAT by anticipating and preparing the patients for relevant potential challenges (10, 11). Several studies have evaluated multimodal prehabilitation programs combining exercise, nutritional support, and psychological interventions, demonstrating that prehabilitation during NAT is feasible and safe and may lead to faster postoperative recovery, reduced treatment-related side effects, and improvements in physical and psychological outcomes (10–16). In patients with esophagogastric cancer, improvements in cardiopulmonary fitness and muscle mass have been observed, along with maintenance of physical performance (12, 13). These changes may enhance treatment response and tolerance and reduce the risk of postoperative complications, although high-quality evidence remains limited (10–13, 15, 17, 18). In addition, prehabilitation has been associated with improved QoL and greater patient empowerment by enabling individuals to take an active role in their own cancer care (19).

Integrative Oncology (IO) is defined as a subspecialty of cancer care combining conventional oncological treatments with complementary approaches with the aim to support the patients physical, emotional, and spiritual well-being (18–27). These interventions are integrated to strengthen patients’ overall condition, reduce symptom burden, enhance treatment tolerance, and improve well-being and QoL (24–27). This holistic and evidence-based approach is increasingly acknowledged in major clinical guidelines, such as those of the American Society of Clinical Oncology (ASCO) and the Society for Integrative Oncology, supplementing standard cancer therapy (20–22).

The integration of a holistic prehabilitation approach into cancer care can be implemented via a prehabilitative IO Day Clinic based on Mind-Body Medicine (MBM). MBM helps manage sleep disturbances and other psychosocial symptoms in cancer survivors (28, 29), and enhances physical, emotional, cognitive, and social functioning, alleviates fatigue, pain, anxiety, and depression, and supports coping and overall life satisfaction (30). Programs combining mindfulness, yoga, exercise, nutrition counseling, cognitive restructuring, and self-help strategies have improved global QoL and reduced stress, anxiety, and depression, particularly in breast cancer patients undergoing chemotherapy (31).

Building on this foundation, the PRehabilitation Integrative MEdicine Day Clinic (PRIME-DC) study aimed to evaluate the feasibility of a multimodal IO program for patients undergoing NAT prior to surgery, with the objective of mitigating deterioration in physical function and QoL and maintaining or improving mental and physical health parameters during NAT across different tumor entities. It also provides insights whether an IO prehabilitation day clinic can be optimized within the context of cancer treatment and recovery.

## 2 Materials and methods

### 2.1 Overview

This mono-center, single-arm interventional study aimed to assess the feasibility of implementing a prehabilitation day clinic program for cancer patients at Ulm University Hospital, a maximum care provider in Baden-Württemberg, Germany. This study was designed as a feasibility trial rather than an efficacy study targeting tumor-specific outcomes. The aim was not to demonstrate uniform clinical effectiveness across tumor types, but to evaluate the feasibility, adherence, acceptability, and implementation of a multimodal IO prehabilitation program within a real-world oncologic setting. Feasibility studies designed for pragmatic trials aim to investigate real-world applicability, including procedures such as eligibility and recruitment that reflect usual care conditions, before progressing to disease-specific trials (32). This investigation was carried out and documented in compliance with the Consolidated Standards of Reporting Trials (CONSORT) guidelines, the extension for pilot and feasibility trials, and the template for intervention description and replication (TIDieR) checklist (33–35).

During the preparation of this work the authors used ChatGPT (OpenAI, San Francisco, CA, USA) and DeepL Write (DeepL SE, Cologne, Germany) in order to assist with language editing. After using these tools, the authors reviewed and edited the content as needed and take full responsibility for the content of the published article.

### 2.2 Participants

All cancer patients with abdominal tumors, and breast cancers undergoing treatment at the Comprehensive Cancer Center Ulm were evaluated for eligibility for the study. Patients were required to be scheduled for, currently receiving, or to have recently completed NAT prior to planned curative surgery. All patients initiated their planned therapy during the intervention period. Further inclusion criteria were: being at least 18 years old, and having the capacity to comprehend the nature and individual implications of the clinical trial. Patients were excluded from the study if they had an American Society of Anesthesiologists physical status classification of greater than three, if they were unable to walk unassisted, or if they were currently participating in another study that could potentially affect the intervention and outcomes of this trial. Only patients who have provided informed consent were included in the study.

A maximum of 15 patients were enrolled during every eight-week cycle. Patients who withdrew their informed consent and subsequently dropped out were not replaced since the program was conducted in a closed-group setting. For reasons of traceability, the attendance of each patient was recorded on each day of the day clinic.

### 2.3 Intervention

The day clinic routine which comprised an IO approach, was conducted at the University Hospital Ulm in person on a weekly basis, on Mondays. The total duration of the intervention was eight weeks, and each session lasted 6.5 hours. This eight-week program was offered on three separate times. The intervention course of actions is shown in **Figure 1**. The program was delivered in a multidisciplinary day-care setting with group-based sessions. The program was based on the Essener model (Department of Internal and Integrative Medicine, Kliniken Essen-Mitte, Faculty of Medicine, University of Duisburg-Essen, Essen, Germany) which integrates elements of the MBM model by Herbert Benson (Harvard Medical School) and the Mindfulness-Based Stress Reduction program by Jon Kabat-Zinn (University of Massachusetts) (36). Core components include mindful physical exercise, nutritional counseling, naturopathic self-help techniques, cognitive restructuring, and patient empowerment/self-management training to promote holistic well-being (29, 36). According to this program, the investigated program in this study consisted of the following key elements:

**Figure 1:**
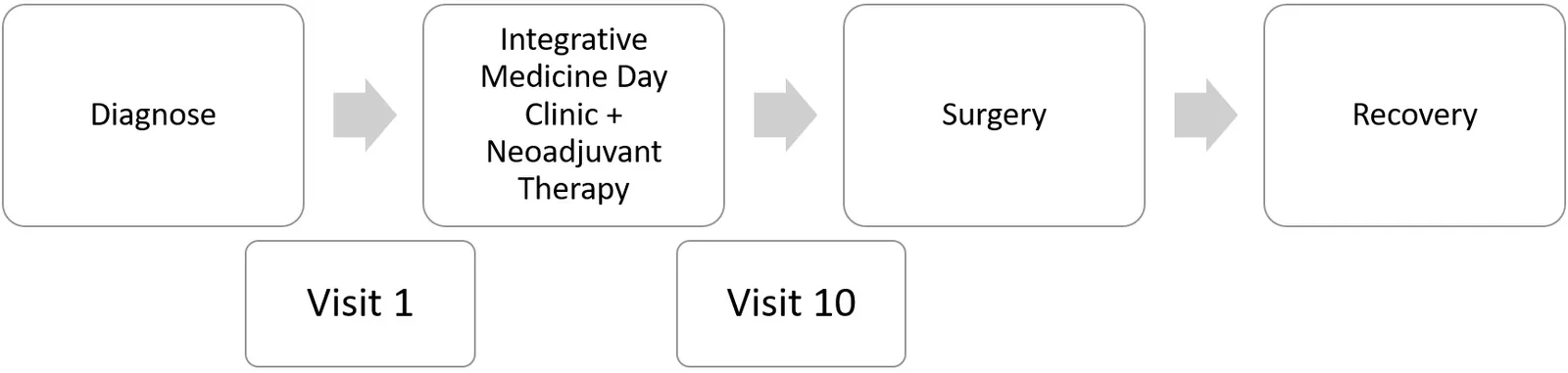
Timeline, the course of events, from diagnosis through intervention to surgery and recovery.

The program was guided by a trained medical doctor and a MBM therapist. Initially, the participants completed the questionnaires, after which a discussion ensued regarding the preceding week. This was followed by a naturopathic consultation conducted by a trained medical doctor. The exercise therapy, conducted by a certified sports therapist, included strength training, aerobic exercises, yoga, and life kinetics. The program was designed according to the FITT principles (frequency, intensity, time, type) as recommended in recent exercise-oncology guidelines and prehabilitation reviews (37, 38): participants attended one supervised 60-minute session per week at moderate intensity. This multimodal approach combined physical training with an educational component to promote correct exercise execution and to provide advice on additional home-based exercises. This session was concluded with a shared integrative lunch. The organic lunch, which was provided by a local caterer, consisted of a salad, a high-protein vegetarian meal and a dessert with fresh fruits.

After lunchtime, the application of a yarrow liver compress (39, 40) was provided by a specially trained integrative nurse, followed by a discussion on MBM topics. The patients were also provided with written information materials. Finally, the last item on the agenda was meditation, for example body scan, breathing meditation, imagination journey. These two parts were also carried out or supervised by the MBM therapist (see **Table 1**). The time slots and contents for these aforementioned components are outlined in **Table 1**. Weeks 1 and 4 deviated from the standard program schedule due to group forming (week 1) and group cooking (week 4). For detailed information regarding the program schedule for each week and the content of the individual program items, please refer to Raff et al., 2023 (24).

**Table 1:** Weekly schedule of day clinic program.

Time (hh:mm)	Content
09:45-10:00	Questionnaire (15 minutes)
10:00-10:30	Week review (30 minutes)
10:30-11:15	Naturopathic consult (45 minutes)
11:15-12:15	Exercise therapy (60 minutes)
12:15-13:00	Lunch (45 minutes)
13:00-14:00	Yarrow liver compress (60 minutes)
14:00-15:45	Introduction to Mind-Body Medicine (105 minutes)
15:45-16:15	Meditation (30 minutes)

with time in 24-hour format (hh:mm).

### 2.4 Outcomes

Main objective of this study was to determine feasibility of prehabilitation during NAT in patients with abdominal or breast tumors. The primary endpoint of feasibility was the patient’s adherence during the day clinic. Adherence was assessed by study personnel and calculated as a ratio of appointments attended out of eight outlined appointments. Attendance was counted if a patient attended at least one of the six program points offered. Secondary endpoints included Patient- and Clinician-Reported Outcomes Measures.

Given the established correlation between heightened mindfulness and diminished pain, fatigue, and psychological distress, the Five-Facet Mindfulness Questionnaire (FFMQ-D) was incorporated as a reliable and valid instrument to assess these focused outcome measures (41, 42). As symptoms and side effects have a significant impact on the QoL of cancer patients, the European Organisation for Research and Treatment of Cancer Quality of Life Questionnaire C30 (EORTC QLQ-C30) was included in the study as a reliable and valid instrument (43–45). Due to the prevalence of anxiety and depression in cancer patients, these aspects were assessed using the validated and reliable Patient health questionnaire 4 (PHQ-4) (46–49). Given the high prevalence of sleep disturbances and their relevance to QoL, sleep quality was assessed using the Pittsburgh Sleep quality Index (PSQI) questionnaire, which has been validated and found to be reliable (16, 50–52). Fatigue, a major challenge for cancer patients undergoing therapy and associated with numerous adverse effects, including diminished QoL, was assessed using the validated and reliable Fatigue Numeric Rating Scale (F-NRS) (53–55). As weight loss is common in cancer patients and is associated with survival and treatment outcomes, Body Mass Index (BMI) was obtained (56–58). The 6-Minute Walking Test (6-MWT), a reliable and valid tool, was employed to assess functional capacity, peak VO2, and global health (59, 60). Additionally, the 1-minute sit-to-stand (STS) test, a reliable and valid tool, was used to evaluate exercise capacity and leg strength (61). Handgrip strength (HGS), recognized as an indicator of overall body strength with known associations to survival outcomes and various biological, functional, and QoL factors, was also incorporated into the study as a valid and reliable instrument (62). This value was set in relation to body weight in kilogram for better comparability (HGS/kg).

A total of 13 visits were conducted by the staff during the intervention period, during which time the endpoints were recorded. The post-intervention measurement was scheduled at Visit 10 (week 10), two weeks after the completion of the 8-week program, to allow assessment of both immediate and short-term retention effects of the multimodal prehabilitation intervention. Prior prehabilitation studies have assessed outcomes few weeks after intervention completion rather than immediately at program end to capture sustained effects and reduce short-term fluctuation due to the final session alone (63). The precise schedule for each visit is outlined in **Table 2**. A protocol-to-publication comparison of the assessed outcomes and variables is presented in **Table 3**.

**Table 2:** Trial visits and documented parameters.

Visit	1	2–9	10a	10b*	11	12	13
Time	Screening	Week 1–8 of prehabi-litation	Post prehabi-litation	1–10 days before surgery	Post-operative day 4	Day of discharge	Post-operative day 30
Inclusion							
Informed consent	X						
Eligibility criteria	X						
Assessments							
Demographics and baseline clinical data	X						
BMI	X		X				X
Questionnaires							
EORTC QLQ-C30	X		X	X	X		X
F-NRS	X	X	X	X	X	X	X
PHQ-4	X		X	X			X
PSQI	X	X**	X	X			X
FFMQ-D	X		X	X			X
Physical examination							
6-MWT	X		X				
HGS/kg	X		X				
1-minute STS test	X		X				
Assessment of postoperative complications					X	X	X
Intensity of analgesic treatment	X				X	X	X
Readmission to hospital							X
Reoperation							X
Time of stay on intensive care unit							X
Duration of hospital stay							X
Adherence to neoadjuvant treatment		X					
Evaluation Day Clinic Satisfaction							X

*only if time between end of prehabilitation and surgery >14 days. **every 4 weeks during prehabilitation. ***if the eight day clinic days are completed. With BMI, Body Mass Index; EORTC QLQ-C30, European Organisation for Research and Treatment of Cancer Quality of Life Questionnaire C30; F-NRS, Fatigue Numeric Rating Scale; PHQ-4, Patient Health Questionnaire 4; PSQI, Pittsburgh Sleep Quality Index; FFMQ-D, Five-Facet Mindfulness Questionnaire; 6-MWT, 6-Minute Walking Test; HGS/kg, Handgrip strength/body weight in kilogram; STS, sit-to-stand.

**Table 3:** Protocol-to-publication comparison of assessed outcomes and variables.

Outcome	Reported in publication	Status/explanation
Anthropometric assessment		
Body Mass Index	✓	Reported in the current publication
Questionnaires		
EORTC QLQ-C30 European Organisation for Research and Treatment of Cancer Quality of Life Questionnaire C30	✓	Reported in the current publication
Patient-Reported Outcomes version of the Common Terminology Criteria for Adverse Events	–	Not available for analysis
Fatigue Numeric Rating Scale	✓	Reported in the current publication
Quality of Recovery-15	–	Not available for analysis
Patient Health Questionnaire 4	✓	Reported in the current publication
Pittsburgh Sleep Quality Index	✓	Reported in the current publication
Five-Facet Mindfulness Questionnaire	✓	Reported in the current publication
Physical examination		
6-Minute Walking Test	✓	Reported in the current publication
Handgrip strength/body weight in kilogram	✓	Reported in the current publication
1-minute sit-to-stand test	✓	Reported in the current publication
Assessment of postoperative complications		
Intensity of analgesic treatment	–	Not available for analysis
Readmission to hospital	–	Not available for analysis
Reoperation	–	Not available for analysis
Time of stay on intensive care unit	–	Not available for analysis
Duration of hospital stay	–	Not available for analysis
Adherence to neoadjuvant treatment	–	Not available for analysis
Evaluation Day Clinic Satisfaction	✓	Reported in the current publication

Furthermore, a satisfaction evaluation questionnaire was administered to patients, querying their level of satisfaction with the day clinic’s implementation, the day clinic in general, and each program component in particular (on a Likert scale from 0 – very satisfied - to 4 - very unsatisfied).

The timeline of the program is shown in **Figure 1**.

### 2.5 Sample size

PRIME-DC was a feasibility study with an exploratory approach. Therefore, no formal sample size calculation was performed. The study population was planned as a minimum number of 18 participants and a maximum of 45 patients to fulfill the organizational needs of the group-based program. For details see Raff et al., 2023 (24).

### 2.6 Data processing

All questionnaires and tests were evaluated for each visit separately in accordance with the methodologies described in the literature and the respective scoring manuals (41, 46, 50, 55, 64).

### 2.7 Statistical analysis

All statistical analyses were conducted using IBM SPSS Statistics, version 30.0. Prior to inferential testing, the normality assumption was assessed using the Kolmogorov–Smirnov test. Given that a substantial proportion of the outcome variables deviated from normality (32 out of 61), all paired comparisons were performed using the Wilcoxon signed-rank test to ensure a consistent nonparametric analytical approach across outcomes. To evaluate the psychological and physical effects of day clinic treatment, data from Visit 1 (V1) were compared with data from Visit 10a (V10a). Only participants with complete paired data for the respective variable at both Visit 1 (V1) and Visit 10a (V10a) were included in each analysis. Missing values were not imputed. Thus, the number of participants included (n) could vary between variables depending on the availability of paired data. The number of patients included in each analysis is listed in **Table 4**, **5**. These values provide transparency regarding the available data and allow the extent of missing data across assessments to be determined.

**Table 4:** Results of the mental health parameters.

Parameter	n	Visit 1 [median (25th percentile/75th percentile)]	Visit 10a [median (25th percentile/75th percentile)]	95% CI	p-value	Effect size (r)
FFMQ-D						
Nonjudging to inner experience	12	32.00 (28.00/34.00)	32.00 (27.75/37.00)	-3.000 – 3.000	0.505	0.19
Describing	11	32.00 (28.00/35.00)	31.00 (29.00/33.00)	-2.500 – 1.000	0.570	0.17
Observing	10	30.00 (28.00/35.25)	31.00 (27.00/35.25)	-2.500 – 1.500	0.394	0.27
Acting with Awareness	11	29.00 (27.00/35.00)	31.00 (28.00/34.00)	-3.000 – 3.000	0.893	0.04
Nonreactivity to inner experience	11	23.00 (22.00/25.00)	25.00 (21.00/27.00)	-2.000 – 3.000	0.635	0.14
Total	10	147.50 (134.50/160.75)	145.00 (135.75/164.25)	-4.000 – 8.000	0.540	0.19
EORTC QLQ-C30						
Global Health status	18	66.67 (50.00/75.00)	50.00 (41.67/66.67	-20.833 – 8.333	0.255	0.27
Physical functioning	18	90.00 (73.33/100.00)	86.67 (83.33/95.00)	-6.667 – 6.667	0.733	0.08
Role functioning	18	66.67 (33.33/100.00)	66.67 (66.67/83.33)	-8.333 – 8.333	0.166	0.33
Emotional functioning	18	66.67 (41.67/83.33)	66.67 (56.25/85.42)	0.000 – 12.500	0.169	0.32
Cognitive functioning	18	91.67 (62.50/100.00)	75.00 (66.67/100.00)	-8.333 – 16.667	0.721	0.08
Social functioning	18	83.33 (50.00/100.00)	66.67 50.00(/100.00)	-16.667 – 8.333	0.888	0.03
Fatigue	18	33.33 (19.44/58.33)	33.33 (30.56/44.44)	-16.667 – 11.111	0.513	0.15
Nausea and vomiting	18	0.00 (0.00/20.83)	0.00 (0.00/20.83)	-8.333-8.333	0.865	0.04
Pain	18	33.33 (0.00/50.00)	16.67 (0.00/33.33)	-25.000-0.000	0.101	0.39
Dyspnoe	18	33.33 (0.00/66.67)	33.33 (0.00/66.67)	-4.974E-13 – 16.667	0.721	0.08
Insomnia	18	33.33 (33.33/66.67)	33.33 (33.33/66.67)	-4.974E-13 – 16.667	0.759	0.07
Appetite loss	18	0.00 (0.00/8.33)	0.00 (0.00/33.33)	0.000 – 33.333	0.160	0.33
Constipation	18	0.00 (0.00/33.33)	0.00 (0.00/33.33)	-16.667 – 16.667	0.943	0.02
Diarrhea	18	16.67 (0.00/75.00)	16.67 (0.00/66.67)	-16.667 – 16.667	0.646	0.11
Financial difficulties	18	0.00 (0.00/33.33)	0.00 (0.00/0.00)	-16.667 – 0.000	0.279	0.26
PHQ-4						
PHQ-4 anxiety	17	1.00 (1.00/2.00)	1.00 (0.50/2.00)	-0.500 – 0.000	0.102	0.40
PHQ-4 depression	17	2.00 (0.50/2.00)	2.00 (1.00/2.00)	-0.500 – 0.500	1.000	0.00
PHQ-4 sum score	17	3.00 (1.50/4.00)	2.00 (1.50/4.00)	-1.000 – 0.500	0.458	0.18
PSQI						
PSQI sum score	10	9.00 (5.50/10.50)	8.00 (6.75/12.00)	-1.500 – 3.000	0.441	0.24
F-NRS						
Fatigue score	18	3.00 (2.00/4.00)	3.00 (2.75/4.00)	-0.500 – 1.000	0.336	0.23

with n, number of analyzed cases; EORTC QLQ-C30, European Organisation for Research and Treatment of Cancer Quality of Life Questionnaire C30; F-NRS, Fatigue Numeric Rating Scale; PHQ-4, Patient Health Questionnaire 4; PSQI, Pittsburgh Sleep Quality Index; FFMQ-D, Five-Facet Mindfulness Questionnaire; CI, Confidence Interval.

**Table 5:** Results of the physical parameter.

Parameter	n	Visit 1 [median (25th percentile/75th percentile)]	Visit 10a [median (25th percentile/75th percentile)]	95% CI	p-value	Effect size (r)
Body weight (in kg)	18	73.00 (62.00/97.00)	73.50 (60.75/98.50)	-1.000 – 1.000	0.722	0.08
BMI	18	23.30 (22.15/32.85)	24.75 (21.62/33.03)	-0.250 – 0.350	0.529	0.15
6-MWT (in m)	19	555.00 (514.54/644.00)	579.00 (555.00/651.00)	2.800 – 42.700	0.15^+^	0.50
HGS/kg^+^	18	0.50 (0.39/0.61)	0.52 (0.41/0.60)	-0.021 – 0.028	0.777	0.07
1-minute STS test (in repetition)	19	26.00 (22.00/34.00)	27.00 (24.99/34.00)	1.000 – 6.000	0.02*^,+^	0.66

*p<0.05. ^+^ adjusted due to multiple testing, with n, number of analyzed cases; BMI, Body Mass Index; 6-MWT, 6-Minute Walking Test; HGS/kg, Handgrip strength/body weight in kilogram; STS, sit-to-stand; kg, kilogram; m, meter; CI, Confidence Interval.

For each analysis, medians, interquartile ranges (IQR; 25th and 75th percentiles), the 95% confidence interval (CI), the p-value, and the effect size (r) were reported. The 95% CI of the physical and psychological tests was calculated using the Hodges–Lehmann estimator. The exploratory significance level was set at α = 0.05. The effect sizes (ES) were calculated as $r=\frac{z}{\sqrt{n}}$, where Z represents the standardized test statistic and n the number of observations included in the analysis. Effect sizes were interpreted according to Cohen’s criteria, with values of 0.10, 0.30, and 0.50 representing small, medium, and large effects, respectively (65). To account for multiple testing, p-values for statistically significant findings were re-evaluated. Where the significance status changed following adjustment, this has been clearly indicated in the results section and interpreted accordingly.

Adherence was calculated using a denominator of eight scheduled appointments for all participants. Missed appointments were recorded as missed and were not rescheduled. For participants who dropped out of the study, all scheduled appointments following withdrawal were classified as missed. The 95% CI of adherence was determined using the Clopper-Pearson method.

## 3 Results

Patient enrollment and characteristics: Between 09th March 2022 and 24th September 2023, 41 patients were screened for eligibility. Twenty-three patients participated in the study, whereas 17 declined to participate or were ineligible and one couldn’t be contacted because of incorrect address information. The reasons for declining participation or ineligibility included time commitment (n=4), travel distance (n=4), no surgery or already finished (n=2) or no NAT (n=2), language barriers (n=1), lack of transportation options (n=1), concurrent participation in another study and unwillingness to participate in an additional study (n=1), and no specific reason provided (n=2). 19 patients successfully completed the study. These patients are defined as those who participated in the whole interventional period without dropping out or withdrawing, and who attended the final visit. For the patient flow-chart see **Figure 2**.

**Figure 2:**
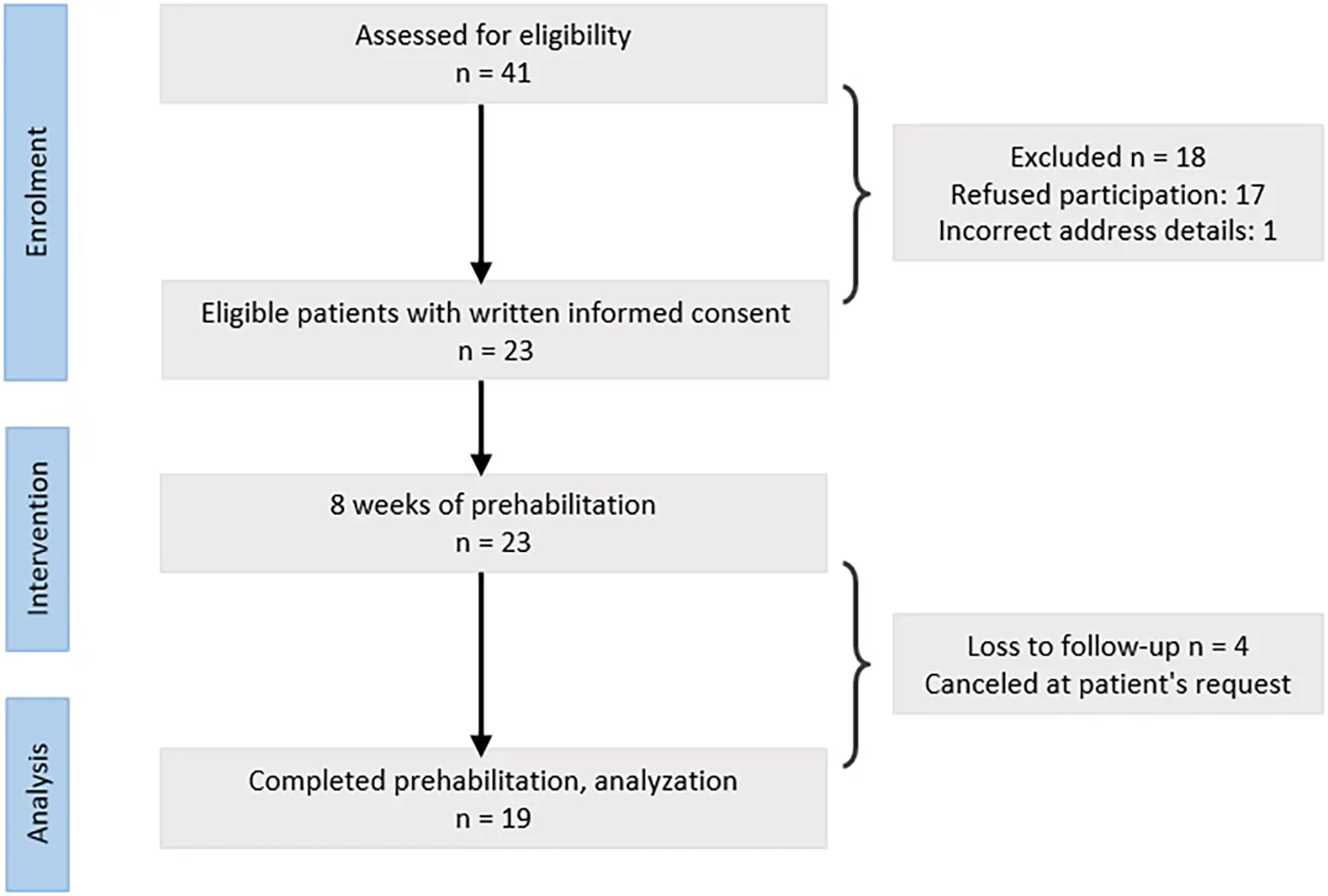
Flow chart, Process from patient enrollment to analysis; n, number of patients.

### 3.1 Feasibility

Fourteen of 23 participants attended at least six of the eight appointments (60.9%, 95% CI = 61%–95%). Therefore, the objective outlined in the study protocol, which was to have 80% of participants complete six of eight appointments, was not met. Five more participants attended fewer than six of the eight sessions, but completed the intervention. Thus, 19 of across all enrolled participants (n = 23) completed the study. The average session attendance for all enrolled participants (n = 23) was 5.65 ± 2.01, while the average attendance for completers (n = 19) was 6.26 ± 1.49. This results in an average session attendance rate of 69%.

The process of recruitment proved to be challenging due to the temporal aspect of the situation. The patient had recently received a diagnosis, and the associated side effects were prevalent during this period. Additionally, the individual had numerous other appointments scheduled prior to undergoing surgery. The reasons for withdrawal or missed sessions also include other appointments due to treatment, side effects, tumor progression, and the time required to participate. Regarding outcome completion, the number of analyzed cases (n) for each outcome and assessment time point is reported in **Table 4**, **5**. No study-related adverse events were observed during the implementation of the program. Exercise sessions were individually adapted to participants’ physical limitations, including restrictions related to ports, injuries, or weakness.

Descriptive data of all participants are outlined in **Table 6**.

**Table 6:** Patient demographics and cancer types of (a) the total number (n=23) and (b) the completers (n=19).

a.		b.	
Characteristics	Value (n, %)	Characteristics	Value (n, %)
Number of Patients	23 (100)	Number of Patients	19 (100)
Sex		Sex	
Male	3 (13.0)	Male	3 (15.79)
Female	20 (87.0)	Female	16 (84.21)
Age in years (± standard deviation)	51,35 (± 13.15)	Age in years (± standard deviation)	49.35 (± 12.83)
Cancer Types		Cancer Types	
Breast carcinoma	17 (73.9)	Breast carcinoma	15 (78.95)
Gastric carcinoma	1 (4.3)	Gastric carcinoma	1 (5.26)
Pancreatic carcinoma	1 (4.3)	Pancreatic carcinoma	1 (5.26)
Rectal carcinoma	4 (17.4)	Rectal carcinoma	2 (10.53)
Therapies		Therapies	
Chemotherapy	22 (95.65)	Chemotherapy	19 (100)
Immunotherapy	11 (47.83)	Immunotherapy	11 (57.89)
Radiotherapy	3 (13.04)	Radiotherapy	2 (10.53)

with n, number of patients and %, in percentage.

### 3.2 Patient-related outcome measures

The majority of patient-related outcome measures demonstrated no statistically detectable within-group changes, or even positive trends, during the prehabilitative IO day clinic, as indicated by mindfulness (FFMQ-D), QoL (EORTC QLQ-C30), anxiety and depression (PHQ-4) or sleep quality (PSQI). Some measures showed a non-significant decline, including global health status, insomnia and fatigue (F-NRS). All results of the mentioned questionnaires are outlined in **Table 4**.

### 3.3 Physical fitness

The HGS/kg showed no statistically detectable within-group changes during the intervention period. Although a moderate effect size was observed for the 6-MWT, the corresponding comparison was no longer statistically significant after adjustment for multiple testing. An exploratory significant within-group improvement in the 1-minute STS test (V1: 26.00 (22.00/34.00), V10a: 27.00 (24.99/34.00), 95% CI = 1.000 – 6.000, p = 0.02, r = 0.66) was observed. The results of the physical parameters are illustrated in the **Table 5**.

### 3.4 BMI

A comparison of the pre-intervention and post-intervention periods revealed no statistically significant difference in BMI among the patients (V1: 23.30 (22.15/32.85) kg/m², V10a: 24.75 (21.62/33.03) kg/m², 95% CI = -0.250 – 0.350, p = 0.529, r = 0.15), contradicting the anticipated decline during NAT.

### 3.5 Satisfaction

Among the 23 subjects enrolled in the study, the evaluation questionnaire was administered only to those who had completed the intervention (n = 19), as the remaining participants declined to participate in subsequent visits. Of the 19 patients who participated in the study, 16 completed the questionnaire and were included in the analysis. However, it should be noted that not all respondents answered every questionnaire item. The mean values and standard deviations for the individual questionnaire items are reported in **Table 7**. **Table 8** presents the frequency distribution of responses for each questionnaire item, based on the number of valid responses available for that item. The corresponding numbers of complete responses are also provided in **Table 8**. The feedback from the satisfaction questionnaire was consistently positive. Both the program and the organization were highly rated, and the participants were very satisfied. There were a few requests for more dates to be organized.

**Table 7:** Results of the Satisfaction Questionnaire of the Integrative Medicine Research Group Ulm on a Likert scale from 0 = very satisfied to 6 = very unsatisfied.

Questions	mean (± sd)
1. How satisfied were you with the registration process at the PRIME Day Clinic?	0,00 ± 0,00
2. How did you like the premises and facilities of the PRIME Day Clinic?	1,88 ± 1,09
3. How did you find the cleanliness at the PRIME Day Clinic?	1,13 ± 0,96
4. How would you rate the friendliness of the staff?	0,00 ± 0,00
5. How satisfied were you with the content of the PRIME Day Clinic program?	0,06 ± 0,25
6. How satisfied were you with the number of appointments at the PRIME Day Clinic?	0,31 ± 0,48
7. How satisfied were you with the scope of the weekly program at the PRIME Day Clinic?	0,13 ± 0,35
8. How pleasant was the atmosphere at the PRIME Day Clinic?	0,06 ± 0,25
9. How would you assess the benefit of participating in the PRIME Day Clinic?	0,13 ± 0,34
10. How satisfied were you with the weekly review at the PRIME Day Clinic?	0,00 ± 0,00
11. How satisfied were you with the integrative medical/naturopathic counseling program at the PRIME Day Clinic?	0,19 ± 0,54
12. How satisfied were you with the exercise program at the PRIME Day Clinic?	0,13 ± 0,34
13. How satisfied were you with the yarrow-liver compress at the PRIME Day Clinic?	0,00 ± 0,00
14. How satisfied were you with the mindfulness and relaxation program at the PRIME Day Clinic?	0,13 ± 0,34
15. How satisfied were you with the nutrition counseling/cooking sessions at the PRIME Day Clinic?	0,57 ± 0,85
16. How satisfied were you with the mindfulness day at the PRIME Day Clinic?	0,14 ± 0,36
17. How satisfied were you overall with the PRIME Day Clinic?	0,00 ± 0,00

with sd, standard deviation; PRIME, PRehabilitation Integrative Medicine; 0.00 ± 0.00 indicates complete agreement among participants. The values shown were recorded after completion of the prehabilitation program.

**Table 8:** Frequency distribution of responses to the Satisfaction Questionnaire.

	Number of responses	Frequency score 0 (%)	Frequency score 1 (%)	Frequency score 2 (%)	Frequency score 3 (%)	Frequency score 4 (%)
Question 1	16	100.0	0.0	0.0	0.0	0.0
Question 2	16	18.8	6.3	43.8	31.3	0.0
Question 3	16	31.3	31.3	31.3	6.3	0.0
Question 4	16	100.0	0.0	0.0	0.0	0.0
Question 5	16	93.8	6.3	0.0	0.0	0.0
Question 6	16	68.8	31.3	0.0	0.0	0.0
Question 7	15	86.7	13.3	0.0	0.0	0.0
Question 8	16	93.8	6.3	0.0	0.0	0.0
Question 9	16	87.5	12.5	0.0	0.0	0.0
Question 10	16	100.0	0.0	0.0	0.0	0.0
Question 11	16	87.5	6.3	6.3	0.0	0.0
Question 12	16	87.5	12.5	0.0	0.0	0.0
Question 13	16	100.0	0.0	0.0	0.0	0.0
Question 14	16	87.5	12.5	0.0	0.0	0.0
Question 15	14	64.3	14.3	21.4	0.0	0.0
Question 16	14	85.7	14.3	0.0	0.0	0.0
Question 17	15	100.0	0.0	0.0	0.0	0.0

The reported frequencies refer to the number of completed responses for each question. The numbering of question refers to **Table 7**.

## 4 Discussion

In this single-center feasibility trial, 23 patients with breast or intraabdominal tumors participated in an 8-week multimodal IO prehabilitation program before, during, or shortly after NAT. The intervention demonstrated high adherence (mean attendance 69%) and 60.9% met the predefined feasibility criteria, with no statistically detectable within-group changes in physical or mental health parameters, and a statistically significant within-group improvement in lower limb functional performance, as assessed by the 1-minute STS test, representing an exploratory finding of this feasibility study. In a setting where declines in health-related QoL and physical fitness are typically observed during NAT, this pattern of non-significant within-group effects and selective improvement is clinically reassuring and aligns with contemporary evidence that multimodal prehabilitation can mitigate functional deterioration during treatment.

Regarding the primary outcome feasibility, 14 of 23 participants completed at least 6 of the 8 predefined intervention sessions, which was the prospectively defined adherence threshold for this study. Although the predefined target of 80% of participants reaching this threshold was not achieved, the findings provide important insights into the practical challenges of implementing a multimodal prehabilitation program during NAT. Reported adherence rates in oncologic prehabilitation programs generally range between 55% and 76%, with higher adherence observed in multimodal interventions that combine exercise, nutritional support and psychological care (15). The mean adherence rate of almost 70% in this study is therefore comparable to those reported in previous multimodal and telehealth-based prehabilitation programs, including findings from systematic reviews of multimodal prehabilitation during NAT (14, 15, 38). Reasons for incomplete adherence included treatment-related factors such as adverse effects, the timing of the intervention within the treatment course, and the required time for participation, as well as competing medical appointments. The aforementioned points also contribute to the challenges associated with the recruitment process. Nevertheless, the results suggest that the program is generally feasible to implement in this patient population while highlighting specific barriers that should be addressed in future studies. The importance of considering comorbidities and daily routines to improve adherence has also been highlighted in implementation research on digital interventions in cancer care (66). Apart from adapting the intervention to patients’ individual physical limitations, no specific safety concerns regarding program implementation were identified. Along with the high satisfaction scores and the absence of study-related adverse events, these findings support the feasibility and acceptability of an IO day-clinic model for prehabilitation in routine cancer care, while acknowledging that the sample size and design are not sufficient to draw firm conclusions about efficacy. The present results align with those of a recent systematic review by Chen et al. (2024), which revealed that prehabilitation for patients undergoing NAT prior to cancer resection is feasible and associated with treatment completion, although the certainty of the evidence is low to moderate (17). In evaluating the feasibility of implementing the program, particular attention must be given to resource requirements, including time, personnel, and organizational effort. Recent implementation-focused studies emphasize that successful integration of prehabilitation into oncologic care depends on several factors. A national survey in gastrointestinal oncology demonstrated that, despite high perceived utility and satisfaction with multimodal prehabilitation programs, implementation remains heterogeneous and is frequently limited by insufficient human resources, coordination challenges, and funding. Similarly, qualitative evaluations of patients with ovarian cancer showed that multimodal prehabilitation is generally well accepted. These evaluations also highlighted the importance of individualized communication, accessibility of healthcare professionals, the amount and timing of information, and including the patient’s social network in discussions as key facilitators of engagement and adherence (67). These findings are further supported by implementation research from adjacent rehabilitation fields, including telemedicine and telerehabilitation in non-oncologic populations (66, 68, 69). Although these studies are not directly comparable to oncologic prehabilitation, they provide valuable insights into cross-cutting determinants of successful implementation, including patient engagement, organizational factors, and adherence. In our study the daily operation of the clinic requires approximately 30 minutes of preparation and an additional 60 minutes for post-session cleaning, representing a moderate but predictable time investment. In week 4, when meals were prepared on site, both pre- and post-session workload increased, requiring additional preparation and follow-up time compared to the other intervention days. In terms of personnel, the program depended on a multidisciplinary team consisting of a physician, a nurse, an MBM therapist, and a sports scientist. From an organizational perspective, the structured nature of the program proved advantageous, as core components were predefined, resulting in minimal preparation time for staff. In addition, the staff were already sufficiently trained, and only minimal additional preparation time was necessary, suggesting that implementation does not impose substantial training demands before the different sessions. As the amount of missing data in this study was quite high, future studies should consider allocating personnel resources to supervise questionnaire completion, answer participants’ questions, remind them to complete all items, and review questionnaires for completeness immediately after completion. This may help reduce missing data and improve data quality. To comprehensively address the question of resource utilization, future analyses should also consider additional factors such as cost-effectiveness, scalability of the program, and potential constraints in routine clinical practice.

Furthermore, we would like to discuss the secondary outcomes in an exploratory manner. Existing unimodal and multimodal prehabilitation programs for cancer patients rarely include complementary therapies, and the integration of naturopathy remains particularly uncommon (70–72), so functional outcomes such as walking capacity are frequently used to evaluate effectiveness of prehabilitation interventions. With regard to functional capacity, findings on the 6-MWT are heterogeneous. A postoperative improvement of 14 m (73) is considered the minimal clinically important difference in a population undergoing abdominal surgery, with other studies defining this threshold as 20 m. Several randomized prehabilitation trials including studies by Gillis et al. (2014), Bousquet-Dion et al. (2018), and Carli et al. (2020) (74–76), as well as the home-based intervention by Brahmbhatt et al. (2020) (77) report improvements exceeding 20 m, indicating clinically relevant gains in 6-MWT distance. A comparison of the control group with the prehabilitation group demonstrates higher rates of clinically meaningful improvement in favor of prehabilitation. This finding is reinforced by a Cochrane meta-analysis by Molenaar et al. (2023) reporting a pooled preoperative improvement of the prehabilitation group by 24.91 m (95% CI = 11.24–38.57), consistent with a clinically relevant effect (71). In the present study, a non-significant but clinically relevant improvement of 29 m was observed. In addition, no changes in hand grip strength were identified within the present cohort or in the reported study (77). Hand grip strength has been associated with overall survival and other clinical and functional outcomes (62). Another important outcome parameter is fatigue. Fatigue is widely regarded as a pivotal metric in formulating assertions concerning persistent fatigue following treatment (78). In the present study, no difference in fatigue was observed between the two assessment timepoints, which contrasts with findings from other studies reporting significant differences (14, 79, 80). One possible explanation for this discrepancy is the smaller sample size in our study and the use of more differentiated questionnaires in previous work. Those studies applied the Functional Assessment of Chronic Illness Therapy (FACIT)-Fatigue Scale and the Multidimensional Fatigue Inventory (MFI), which include more items than our instrument. However, our results are consistent with those of Haller et al. (2021) (31). A key difference between studies reporting significant effects and both our study and that of Haller et al. (2021) (31) lies in the treatment context: in Wu et al. (2021), only 11% of patients received chemotherapy or radiotherapy (14), and in Schricker et al. (2025) not all patients underwent chemotherapy or surgery (79). In contrast, all patients in our study received chemotherapy, with some additionally receiving immunotherapy or radiotherapy before surgery; similarly, all patients in the study by Haller et al. received chemotherapy (31). In the context of this feasibility trial, the absence of significant changes could indicate that patients’ functional status and fatigue remained stable during the intervention period. However, the limited sample size precludes firm conclusions and warrants cautious interpretation. Another outcome parameter of this study was BMI. BMI has been shown to be associated with pathophysiological changes related to the tumor, including altered inflammatory and metabolic signaling, adipokine-driven tumorigenesis, and immune- and metabolism-related molecular signatures in multiple cancer types (81–83), and may also influences breast cancer patient’s QoL with higher BMI linked to worse QoL and slower QoL recovery in breast cancer patients undergoing radiotherapy (84). Conversely, anxiety, depression, fatigue and pain have been associated with weight loss and malnutrition (85). Notably, this study observed no change in BMI, which aligns with the findings of Wu et al. (2021) (14).

The most pronounced functional change was observed in the 1-minute STS test, which is a reliable measure of lower-limb strength and exercise capacity (61). Participants completed significantly more repetitions over the 8-week period, representing an exploratory within-group improvement in lower limb functional performance despite ongoing or recent NAT. This may indicate a clinically relevant benefit, as functional capacity is a key determinant of postoperative outcomes and overall QoL in oncologic patients (86, 87). Although cancer-specific minimal clinically important differences for the 1-minute STS test have not yet been established, thresholds of approximately 2–3 repetitions have been suggested in other clinical populations (88); the magnitude of change observed here therefore appears likely to be meaningful, albeit this interpretation should be made with caution.

Previous work suggests that exercise-focused prehabilitation without psychological support may not substantially change health-related QoL (77), highlighting the need for additional mind–body or psychosocial interventions (89). As this study is based on MBM, modules containing such content were integrated into the multimodal program, yet no significant changes were detected in QoL, mindfulness, sleep, anxiety or depression scores. One possible explanation is that MBM modules were delivered with less intensity and volume than in dedicated MBM group programs that have reported larger psychosocial benefits (31, 79, 90). Nevertheless, the fact that these parameters showed no statistically detectable within-group changes during NAT, a period during which deterioration is often expected, may be considered a favorable observation and could suggest that the intervention has the potential to help prevent typical declines in psychological well-being. However, this interpretation should be made with caution given the small sample size and requires confirmation in larger, controlled studies (31, 91, 92).

Overall, meta-analyses suggest that unimodal and multimodal prehabilitation may reduce intensive care unit length of stay by approximately 0.57 days (28% reduction), although effects on total hospital length of stay and 30-day postoperative complications remain inconsistent (93). Beyond clinical outcomes, qualitative studies have consistently shown that patients value integrative, expert-guided multimodal approaches combining exercise, nutritional counseling, and psychological support rather than isolated interventions (77, 94–96). The present IO day-clinic model aligns with these patient-centered principles by providing structured multidisciplinary support within routine oncologic care pathways and may represent a feasible framework for integrating comprehensive supportive care during cancer treatment. Recent evidence from multimodal prehabilitation trials, including the PREHAB randomized clinical trial and studies evaluating prehabilitation during NAT, further supports the potential value of structured multimodal approaches to preserve functional capacity and improve postoperative recovery (17, 71). However, further comparative and health economic studies are needed to evaluate the effectiveness of the intervention and its impact on cancer-specific outcomes. This will help to improve its transferability into clinical practice (63, 97–99).

It is important to note that the analysis was performed among study completers only, and this aspect should be considered when interpreting the findings. Possible limitations of this study include its single-center design, which may limit the generalizability of the findings to other clinical settings. Since most of the patients in the study were women with breast cancer, the results may not be representative of all tumor and surgical populations. This reflects a common challenge in studies evaluating prehabilitation in patients undergoing NAT, where breast cancer cohorts are frequently overrepresented (100). In addition, selection bias cannot be excluded, as participation may have been influenced by patient motivation and clinical eligibility for a multimodal prehabilitation program. Further limitations comprise the small sample size, the heterogeneity of tumor entities and treatment regimens, the lack of a randomized control group, and the absence of post-surgical outcome analyses. However, several strengths should also be acknowledged. The study was conducted in a real-world clinical setting, reflecting routine care conditions. Furthermore, the intervention was delivered by a structured interdisciplinary team and followed a standardized multimodal protocol, which enhances the internal consistency and reproducibility of the program. As a feasibility study, these characteristics provide valuable insights into the implementation, adherence, and acceptability of a multimodal IO prehabilitation program across a diverse oncologic population. Future studies should incorporate more detailed process evaluations, including adherence to the individual intervention components, comprehensive treatment-related information (e.g., timing and number of NAT cycles), surgical details, documentation of protocol deviations, and data on how the intervention was implemented in the home setting. Such information would facilitate a more comprehensive evaluation of intervention fidelity and help identify factors influencing adherence and outcomes. Overall the findings lay an important foundation for future randomized controlled trials with larger sample sizes to evaluate clinical efficacy, perioperative outcomes, and cost-effectiveness of such interventions.

This feasibility study demonstrates that a multimodal IO day-clinic prehabilitation program can be implemented during NAT in a real-world oncologic setting, with acceptable recruitment, high adherence, and patient satisfaction. The findings indicate that maintaining physical and psychological health during this intensive treatment period may be achievable; however, these observations should be interpreted as preliminary and require confirmation in larger controlled studies. The study also identified relevant implementation challenges, including treatment-related side effects, competing medical appointments, and organizational barriers that may affect participation. Future research should therefore focus on optimizing intervention delivery, evaluating implementation strategies, and assessing resource requirements, while larger randomized studies are needed to determine the clinical efficacy, perioperative effects, and cost-effectiveness of integrative prehabilitation programs. Future studies should carefully consider these identified limitations and implementation challenges to optimize study design, improve feasibility and adherence, strengthen the evidence base, and enable more robust conclusions regarding the clinical impact and integration of integrative prehabilitation into standard oncologic care pathways.

## Data Availability

The datasets presented in this article are not readily available because of e.g. ethical restrictions. Requests to access the datasets should be directed to svenja.klaus-karwisch@uniklinik-ulm.de.
